# 945. Bacteremia in Patients with Solid Tumors: Epidemiology, Clinical Features and Risk Factors for Mortality. Results from a Multicenter Study in Argentina

**DOI:** 10.1093/ofid/ofab466.1140

**Published:** 2021-12-04

**Authors:** Patricia E Costantini, Diego Torres, Miguel Dictar, Andrea Nenna, Alejandra Valledor, Rosana Jordán, Ana Laborde, Sandra Lambert, José Benso, Alberto Carena, Martín Luck, Agustina Racioppi, Laura Barcan, María José Eusebio, María Luz Gonzalez Ibañez, Lucas Tula, Fernando Pasteran, Alejandra Corso, Melina Rapoport, Marcelo Bronzi, Federico Nicola, Sandra Valle, María Laura Chaves, Graciela Greco, Renata Monge, María Cristina García Damiano, Miriam Blanco, Fabián Herrera

**Affiliations:** 1 Instituto de Oncología Ángel H Roffo University of Buenos Aires, Buenos Aires, Ciudad Autonoma de Buenos Aires, Argentina; 2 Centro de Educación Médica e Investigaciones Clínicas, CEMIC, Buenos Aires, Ciudad Autonoma de Buenos Aires, Argentina; 3 Instituto Alexander Fleming, Buenos Aires, Buenos Aires, Ciudad Autonoma de Buenos Aires, Argentina; 4 Hospital Municipal de Oncología Marie Curie, Buenos Aires, Ciudad Autonoma de Buenos Aires, Argentina; 5 Hospital Italiano de Buenos Aires, Buenos Aires, Ciudad Autonoma de Buenos Aires, Argentina; 6 Hospital Británico de Buenos Aires, Buenos Aires, Ciudad Autonoma de Buenos Aires, Argentina; 7 FUNDALEU, Buenos Aires, Ciudad Autonoma de Buenos Aires, Argentina; 8 Hospital El Cruce, Florencio Varela, Buenos Aires, Argentina; 9 Hospital Italiano de San Justo, San Justp, Buenos Aires, Argentina; 10 ANLIS-Malbrán, Buenos Aires, Ciudad Autonoma de Buenos Aires, Argentina

## Abstract

**Background:**

Current information regarding bacteriemia in patients with solid tumors is scarce

**Methods:**

To assess the etiology, clinical features and outcome in patients with solid tumors and bacteremia, we carried out a prospective multicenter study. Episodes of bacteriemia in adult cancer patients in 9 centers, from May 2014 to February 2021, were recorded. To identify factors associated with 30-day mortality, variables with p < 0.05 in univariate analysis were included in a logistic regression model for multivariate analysis

**Results:**

Three hundred and thirty-two episodes of bacteremia were included, with 51% being women (mean age 59). The state of underlying disease was: recent diagnosis 27%, remission 27%, relapsed 29% and refractory 17%. Seventy-three percent had received chemotherapy in the last 30 days, 25% were receiving steroids. Neutropenia was present in 23% (mean duration 3 days). The most frequent sources were: abdominal 39%, urinary tract 21%, respiratory 15%, catheter 10% and skin and soft tissue 9%. The microorganisms were: Gram negative bacilli (GNB) 67% (Enterobacterales 84%), Gram positive cocci 36% (*Staphylococcus aureus* 33%) and polimicorbial 11%; 20% were multidrug resistant organisms (MDR-O), being 88% of them GNB (MDR-GNB). ESBL and KPC carbapenemase producing were the most frequent mechanisms of resistance. Mortality at day 7 and day 30 was 16% and 27%, respectively. In the univariate analysis, the risk factors for 30-day mortality were Charlson index, refractory underlying disease, use of steroids, polimicrobial bacteremia, *Staphylococcus aureus*, GNB resistant to carbapenems, APACHE and Pitt scores, hypotension, respiratory source and ICU admission. In multivariate analysis, risk factors for 30-day mortality were refractory underlying disease, GNB resistant to carbapenems and ICU admission, while 7-day clinical response was associated with lower mortality

**Conclusion:**

Bacteremia is a serious complication in cancer patients, with high mortality. The state of underlying disease, infection caused by GNB resistant to carbapenems, and the severity of presentation are associated with increased mortality. Our results stress the importance of infection control measures and antibiotic stewardship to prevent colonization with MDR-O

**Disclosures:**

**All Authors**: No reported disclosures

